# Evaluation of the Effect of IL‐1 Antagonists on Pituitary Function

**DOI:** 10.1155/ije/8796993

**Published:** 2026-01-11

**Authors:** Fadime Aktas Koc, Baris Sariakcali, Ali Sahin

**Affiliations:** ^1^ Department of Internal Medicine, Sivas Numune Hospital, Sivas, 58140, Türkiye, saglik.gov.tr; ^2^ Department of Endocrinology, Sivas Cumhuriyet University, Sivas, 58140, Türkiye, cumhuriyet.edu.tr; ^3^ Department of Rheumatology, Sivas Cumhuriyet University, Sivas, 58140, Türkiye, cumhuriyet.edu.tr

**Keywords:** endocrinology, familial Mediterranean fever (FMF), hormonal levels, IL-1 antagonists, pituitary function

## Abstract

**Background:**

Familial Mediterranean fever (FMF) is a hereditary autoinflammatory disease frequently observed in populations along the Eastern Mediterranean coast, characterized by recurrent fever, abdominal pain, and joint inflammation. The disease results from mutations in the MEFV gene, which plays a critical role in regulating IL‐1β secretion. Mutations in pyrin lead to uncontrolled IL‐1β release, driving FMF’s inflammatory symptoms. IL‐1 inhibitors, such as anakinra, rilonacept, and canakinumab, have been introduced as adjunctive treatments. This paper aims to investigate the effects of IL‐1 inhibitors on pituitary functions in FMF patients.

**Methods:**

The study was conducted at Sivas Cumhuriyet University Hospital and included patients who had been using IL‐1 inhibitors for at least 6 months. The control group consisted of patients receiving colchicine treatment only. Blood samples were collected to measure various pituitary and endocrine hormones. Patients with conditions like corticosteroid use, cancer, or hemodialysis were excluded. Hormonal levels were analyzed, and cortisol‐deficient patients underwent a Synacthen test.

**Results:**

No significant differences were found in TSH, ACTH, cortisol, LH, estradiol, IGF‐1, or PRL levels between the groups. However, differences were noted in FSH, total testosterone, and GH levels, with higher FSH and GH in the control group and higher testosterone in the experimental group.

**Conclusions:**

Although IL‐1 plays a role in hormone secretion pathways, further studies are needed to better understand the effect of IL‐1 antagonists on pituitary function, as no significant adrenal or pituitary insufficiencies were observed.

## 1. Introduction

The pituitary gland is located within a structure called the sella turcica at the base of the skull [1]. Hormones secreted by the anterior pituitary include adrenocorticotropic hormone (ACTH), prolactin (PRL), growth hormone (GH), thyroid‐stimulating hormone (TSH), follicle‐stimulating hormone (FSH), luteinizing hormone (LH), and melanocyte‐stimulating hormone (MSH) [2]. The posterior pituitary hormones are vasopressin (antidiuretic hormone, ADH, and AVP) and oxytocin [1]. Pituitary hormones play a crucial role in maintaining body homeostasis and regulating the functions of other endocrine glands. These hormones are stimulated or inhibited through a feedback system, which can be positive, negative, or multihormonal [3].

Hypopituitarism is defined as a deficiency of one or more hormones produced by the pituitary gland [3]. It may develop due to congenital defects or acquired causes. Traumatic causes, including pituitary adenoma surgery, are common causes of pituitary insufficiency. Other conditions that can lead to pituitary insufficiency include pituitary adenomas, peri pituitary tumors, Sheehan syndrome, pituitary apoplexy, aneurysms, infections, inflammatory conditions, various drugs, and more [4].

In a study conducted by Regal et al. in Northwestern Spain, the prevalence of hypopituitarism was reported to be 45/100,000, with an incidence of approximately 4/100,000/year, and multiple hormone deficiencies were found in about half of the cases [5].

The clinical presentation of pituitary insufficiency depends on which hormone is deficient and the degree of deficiency. It varies according to the age of onset, duration, and underlying cause of the disease [5]. Hypopituitarism usually progresses slowly, making hormone deficiencies easy to overlook in the early stages. When diagnosing, it is crucial to suspect patients presenting with symptoms of hormone deficiencies and to take a thorough history of hypothalamic–pituitary diseases. Therefore, patients with a history of hypothalamic and pituitary masses, surgery, and radiotherapy of the pituitary and neighboring regions, head trauma leading to loss of consciousness and/or hospitalization, meningitis and encephalitis, severe bleeding during childbirth, and/or absence of lactation are considered at risk for pituitary insufficiency. They should be evaluated with baseline hormone levels and, if necessary, dynamic endocrine tests [4].

Familial Mediterranean fever (FMF) is a familial autoinflammatory syndrome characterized by recurrent fever, abdominal pain, arthralgia, peritonitis, pleuritis, and erysipelas‐like skin lesions, and it is most commonly seen in populations along the Eastern Mediterranean coast [6].

FMF is generally inherited in an autosomal recessive pattern and is caused by mutations in the MEFV gene, which is located on chromosome 16 and consists of 10 exons [7]. More than 300 variants have been reported in the MEFV gene to date. The majority of cases involve founder mutations located in exon 10 of the MEFV gene, which encodes the C‐terminal B30.2 domain of pyrin. These mutations are M694V, M680I, M694I, and V726A [8]. Among Turkish patients, after M694V, M680I and V726A are the most common mutations [9].

The most impediment criterion for diagnosing FMF is clinical findings. FMF should be considered in the differential diagnosis of patients with complaints of abdominal pain, arthritis, joint pain, and fever, as well as a history of emergency surgeries due to acute abdomen [9]. FMF diagnosis is based on clinical features, supported by ethnic background and family history. Genetic testing for FMF is used to support the diagnosis in patients who meet the clinical criteria. In patients with a family history and clinical attacks, an FMF genetic test should be requested. If the genetic test result is negative or indicates carrier status, but the patient has clear FMF‐like symptoms and a family history, colchicine treatment is administered. If the patient benefits from the treatment, FMF diagnosis is strongly considered despite the negative genetic result [10].

The primary goal in treating FMF is to effectively manage acute attacks, reduce chronic and subclinical inflammation, prevent complications, and ensure patients maintain an optimal quality of life [11]. The cornerstone of FMF management is the daily administration of oral colchicine, which has proven efficacy in preventing both acute attacks and the development of serum amyloid A amyloidosis. However, in cases where patients experience frequent attacks—more than one every three months—or exhibit persistently elevated inflammatory markers during attack‐free periods, it is essential to increase the colchicine dosage [12].

Colchicine resistance is defined as using the maximum tolerated dose of colchicine and experiencing one or more attacks per month within the past 6 months [8]. Alternative treatment strategies should be considered for patients with colchicine resistance [13]. Interleukin‐1 (IL‐1) is a central mediator of immunity and inflammation. It affects the entire body. The pathophysiological functions of IL‐1 include immune system activation, including inflammation, thymocyte maturation, and T helper 2 cell proliferation, bone metabolism, fever generation, and stimulation of the hypothalamic–pituitary–adrenal (HPA) axis. IL‐1 is important for maintaining body homeostasis and controlling the immune system by the central nervous system [14] Pyrin is encoded by the MEFV gene. Regulation of IL‐1β secretion is a key function of pyrin. Pathogenic variants in the MEFV gene cause overstimulation of the pyrin inflammasome and caspase 1. This results in excessive IL‐1β secretion. Following the discovery of pyrin’s effect on IL‐1 synthesis, the use of anti‐IL‐1 agents has increased in colchicine‐resistant patients [8]. Anti‐IL‐1 therapy reduces the frequency of relapses and the risk of amyloidosis. It also improves patients′ quality of life [15].

Anakinra is the first drug used for this purpose. It is a recombinant, nonglycosylated human IL‐1 receptor antagonist. It binds to the IL‐1 receptor, preventing its interaction with IL‐1 alpha and IL‐1 beta, thereby reducing the activity of these interleukins [15]. The most common side effect of anakinra is a reaction at the injection site. An increase in infection rates, particularly in the upper respiratory tract, has been observed. Side effects include interstitial pneumonia, neutropenia, hypertension, headache, arthralgia, and gastrointestinal symptoms. High rates of severe and invasive necrotizing fasciitis, sepsis, infections leading to tissue abscesses, and tuberculosis reactivation with group A streptococci have been associated with anakinra treatment [16, 17]. In contrast, the most common side effect of canakinumab, which has a longer half‐life and is administered monthly subcutaneously, is upper respiratory tract infection, followed by urinary tract infection, pneumonia, latent tuberculosis, and lymphadenitis [18]. Reactions at the injection site, headaches, dizziness, and hypersensitivity reactions may occur [19].

What sets this paper apart is its focus on investigating the specific effects of IL‐1 antagonists on pituitary hormonal axes, an area that has not been extensively explored in the literature. By evaluating this interaction, the study seeks to provide novel insights into the broader systemic effect of IL‐1 inhibition in FMF patients, contributing valuable knowledge to both endocrinology and autoinflammatory disease management.

## 2. Methods

This paper was conducted in the Department of Internal Medicine, Division of Endocrinology and Metabolism, and Division of Rheumatology, at Sivas Cumhuriyet University Faculty of Medicine Research and Application Hospital in Türkiye. This paper was approved by the Ethics Committee of Sivas Cumhuriyet University Faculty of Medicine on 16.02.2021 (Decision No. 2021‐02/05). The study was carried out in accordance with the Declaration of Helsinki. It was supported by Scientific Research Projects Coordination Unit of Sivas Cumhuriyet University (CUBAP) as project number T‐2021‐930.

**Figure 1:**
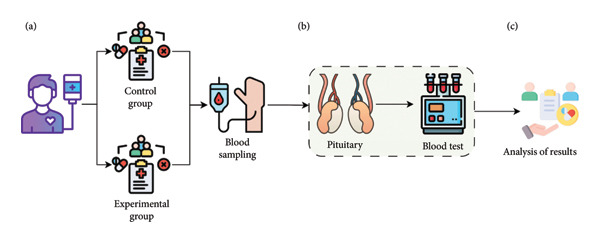
Study design illustrating the effect of IL‐1 antagonists on pituitary function. (a) Blood samples were collected from the experimental group receiving IL‐1 antagonists and the control group not receiving IL‐1 antagonists. (b) ACTH, basal cortisol, TSH, free T4, free T3, anti‐thyroglobulin antibodies, anti‐microsomal antibodies, FSH, LH, PRL, GH, and IGF‐1 levels were analyzed in all patients. Estradiol levels were assessed in female patients, and total testosterone (T TEST) levels were assessed in male patients. (c) The results were then analyzed.

According to the results of the power analysis, the total sample size required for a moderate effect size (*d* = 0.5), 5% significance level (*α* = 0.05\alpha = 0.05), and 80% power (1‐β = 0.81) was calculated as approximately 64 individuals. Since current sample size is 90 people (experimental group: 45, control group: 45), this paper has a sufficient sample in terms of power analysis. This means that the statistical significance is sufficient to recognize a moderate effect. The flowchart of the study is illustrated in Figure 1.

### 2.1. Inclusion Criteria for Patients

This prospective study included patients over the age of 18 who had been using IL‐1 antagonists for at least six months and who voluntarily agreed to participate in the study by signing informed consent forms. Both groups of patients, those using and not using IL‐1 antagonists, were also receiving colchicine treatment. Patients from the city of Sivas who met the inclusion criteria and volunteered to participate were included in the study.

### 2.2. Exclusion Criteria for Patients

Patients younger than 18 years old, those using IL‐1 antagonists for less than 6 months, patients receiving any corticosteroids or related drugs, cancer patients, severely cachectic patients, and patients on hemodialysis were excluded from the study. Care was taken to ensure that the patients included in the study did not have additional comorbidities that would affect hormone levels.

### 2.3. Variables Evaluated

The variables evaluated in this paper included ACTH, basal cortisol, TSH, free T4, free T3, anti‐thyroglobulin antibodies, anti‐microsomal antibodies, FSH, LH, PRL, GH, and IGF‐1 in all patients. Estradiol levels were evaluated in female patients, and total testosterone (T TEST) levels were evaluated in male patients. Age, gender, and the status of whether patients were using IL‐1 antagonists or not were also recorded.

### 2.4. Data Collection

Morning blood samples were collected from each patient, with 10 mL drawn into one EDTA hemogram tube and three serum separator tubes. The blood collection process took place between 26.08.2021 and 03.12.2022. The collected blood samples were analyzed at the Biochemistry Laboratory of Sivas Cumhuriyet University Faculty of Medicine Research and Application Hospital using the Roche cobas 23 e801 device. Samples were centrifuged at 4000 rpm for 10 min in a refrigerated centrifuge, and the ECLIA method was used for analysis. Biochemistry results were accessed via the hospital automation system using the SISOFT program. IL‐1 antagonists are used for patients who do not respond or cannot tolerate colchicine. Therefore, they are not very frequently used treatments. When we look at our country in general, Sivas Cumhuriyet University Medical Faculty Research and Application Hospital is one of the centers where IL‐1 antagonists are used the most. Despite this, patients were not matched in terms of age, gender, and disease severity due to the low number of patients who met the inclusion criteria and volunteered to participate in the study using IL‐1 antagonists. The reference ranges for the analyzed parameters (ACTH, basal cortisol, TSH, free T4, free T3, anti‐thyroglobulin antibodies, anti‐microsomal antibodies, FSH, LH, PRL, GH, IGF‐1, estradiol, and T TEST) were determined based on the test guide of the Biochemistry Laboratory at Sivas Cumhuriyet University Faculty of Medicine Hospital. The reference ranges for IGF‐1 and GH were adjusted according to age and gender (Figure 1).

### 2.5. Statistical Analysis

The statistical analyses used in this paper were carefully selected to test the research hypotheses and assess the relationships between the variables. First, descriptive statistics (mean, standard deviation, frequency, and percentage distributions) were used to determine the general distribution and basic characteristics of the data. The *t*‐test for independent samples was used to test for differences in means between two independent groups. This test was chosen because the variables were continuous, the groups were independent of each other, and the assumption of normal distribution was met. Since continuous variables such as hormone levels had to be compared in the study, parametric tests were preferred. In addition, the chi‐square test was used to analyze the relationships between categorical variables. This test was used to examine how hormone levels were distributed in the experimental and control groups according to specific reference ranges (low, normal, and high). Normality analysis was conducted to assess whether the hormone levels followed a normal distribution. These analyses were performed to determine the suitability of parametric tests for evaluating the effects of IL‐1 antagonists on hormone levels, to detect statistically significant differences between the experimental and control groups, and to reliably interpret the research results.

## 3. Results

The study included 90 patients diagnosed with FMF who applied to the Department of Internal Medicine, Division of Endocrinology and Metabolism, and Division of Rheumatology at Sivas Cumhuriyet University Faculty of Medicine Research and Application Hospital. Of these, 45 patients (24 women, 21 men) had been using IL‐1 antagonists for at least 6 months. The control group consisted of 45 patients (31 women, 14 men) diagnosed with FMF but not using IL‐1 antagonists.

The basal hormone levels of the patients were evaluated according to the reference ranges provided by the Biochemistry Laboratory of Sivas Cumhuriyet University Faculty of Medicine Research and Application Hospital. IGF‐1 and GH results were assessed based on age ranges.

According to Table 1, TSH levels were within the normal range for patients using anakinra (*n* = 13) and canakinumab (*n* = 28) in the experimental group, as well as for those using colchicine (*n* = 43) in the control group. Similarly, FT4 levels were observed to be in the normal range for patients in the experimental group using anakinra (*n* = 14) and canakinumab (*n* = 30), as well as for colchicine users (*n* = 41) in the control group. Furthermore, FT3 levels were within the normal range for patients using anakinra (*n* = 15) and canakinumab (*n* = 30) in the experimental group and for those using colchicine (*n* = 45) in the control group.

**Table 1:** Comparison of thyroid function tests according to the reference interval of measurements.

Thyroid function tests						
Value range			Low < 0.27	Medium = 0.27−4.2	High > 4.2	Total
TSH	Experimental group	Anakinra	0 (0.0%)	13 (15.5%)	2 (40.0%)	15 (16.7%)
		Canakinumab	0 (0.0%)	28 (33.3)	2 (40.0%)	30 (33.3%)
	Control group	Colchicine	1 (100.0%)	43 (51.2%)	1 (20.0%)	45 (50.0%)
Total			1 (100.0%)	84 (100%)	5 (100%)	90 (100.0%)

**Value range**			**Low < 0.93**	**Medium = 0.93–1.7**	**Total**	

FT4	Experimental group	Anakinra	1 (20.0%)	14 (16.5%)	15 (16.7%)	
		Canakinumab	0 (0.0%)	30 (35.3%)	30 (33.3%)	
	Control group	Colchicine	4 (80.0%)	41 (48.2%)	45 (50.0%)	
Total			5 (100%)	85 (100%)	90 (100.0%)	

**Value range**			**Medium = 0.93–1.7**	**Total**		

FT3	Experimental group	Anakinra	15 (16.7%)	15 (16.7%)		
		Canakinumab	30 (33.3%)	30 (33.3%)		
	Control group	Colchicine	45 (50.0%)	45 (100.0%)		
Total			90 (100.0%)	90 (100.0%)		

According to Table 2, FSH levels were within the normal range for patients using anakinra (*n* = 14) and canakinumab (*n* = 29) in the experimental group, as well as for those using colchicine (*n* = 37) in the control group. Likewise, LH levels were in the normal range for patients using anakinra (*n* = 14) and canakinumab (*n* = 30) in the experimental group and for those using colchicine (*n* = 45) in the control group. Moreover, estradiol levels were within the normal range for patients using anakinra (*n* = 5) and canakinumab (*n* = 15) in the experimental group, as well as for colchicine users (*n* = 24) in the control group. Additionally, T TEST levels were found to be in the normal range for patients using anakinra (*n* = 7) and canakinumab (*n* = 12) in the experimental group and for those using colchicine (*n* = 13) in the control group.

**Table 2:** Comparison of gonadotropin, estradiol, and total testosterone levels according to the reference interval of measurements.

Gonadotropins, estradiol, and total testosterone						
Value range			Low	Medium	High	Total
FSH	Experimental group	Anakinra	1 (33.3%)	14 (17.5%)	0 (0.0%)	15 (16.7%)
		Canakinumab	0 (0.0%)	29 (3.6, 3.3)	1 (14.3)	30 (33.3%)
	Control group	Colchicine	2 (66.7%)	37 (46.3%)	6 (13.6%)	45 (50.0%)
Total			3 (100.0%)	80 (100.0%)	7 (100.0%)	90 (100.0%)

**Value range**			**Medium**	**High**	**Total**	

LH	Experimental group	Anakinra	14 (16.1%)	1 (33.3%)	15 (16.7%)	
		Canakinumab	28 (32.2%)	2 (66.7)	30 (33.3)	
	Control group	Colchicine	45 (51.7%)	0 (0.0%)	45 (100.0%)	
Total			87 (100.0%)	3 (100.0%)	90 (100.0%)	
Estradiol	Experimental group	Anakinra	0 (0.0%)	5 (11.4%)	3 (42.9%)	8 (14.5%)
		Canakinumab	1 (25.0)	15 (34.1)	0 (0.0)	16 (29.1)
	Control group	Colchicine	3 (75.0%)	24 (54.5%)	4 (57.1%)	31 (56.4%)
Total			4 (100.0%)	44 (100.0%)	7 (100.0%)	55 (100.0%)

**Value range**			**Low**	**Medium**	**High**	**Total**

*T* test	Experimental group	Anakinra	0 (0.0%)	7 (21.9%)	0 (0.0%)	7 (20.0%)
		Canakinumab	1 (50.0)	12 (37.5)	1 (100.0%)	14 (40.0)
	Control group	Colchicine	1 (50.0%)	13 (40.6%)	0 (0.0%)	14 (40.0%)
Total			2 (100.0%)	32 (100.0%)	1 (100.0%)	35 (100.0%)

According to Table 3, ACTH levels were within the normal range for patients using anakinra (*n* = 15) and canakinumab (*n* = 30) in the experimental group, as well as for those using colchicine (*n* = 41) in the control group. Similarly, cortisol levels were found to be in the normal range for patients using anakinra (*n* = 11) and canakinumab (*n* = 25) in the experimental group, as well as for those using colchicine (*n* = 37) in the control group. In assessing cortisol results, values between 7 and 20 μg/dL were considered normal, while values below 7 μg/dL were deemed affected, warranting further investigation for adrenal and pituitary insufficiency. Consequently, a Synacthen test was conducted in 14 patients with cortisol values below 7 μg/dL, revealing peak cortisol levels of 18 μg/dL, thereby excluding adrenal insufficiency in these patients.

**Table 3:** Comparison of adrenocorticotropic hormone and cortisol levels according to the reference interval of measurements.

Adrenocorticotropic hormone and cortisol						
Value range			Low < 7.2	Medium = 7.2–63.3		Total
ACTH	Experimental group	Anakinra	0 (0.0%)	15 (17.4%)		15 (16.7%)
		Canakinumab	0 (0.0%)	30 (34.9%)		30 (33.3%)
	Control group	Colchicine	4 (100.0%)	41 (47.7%)		45 (50.0%)
Total			4 (100.0%)	86 (100.0%)		90 (100.0%)

**Value range**			**Low < 7**	**Medium = 7–20**	**High > 20**	**Total**

Cortisol	Experimental group	Anakinra	4 (28.6%)	11 (15.1%)	0 (0.0%)	15 (16.7%)
		Canakinumab	4 (28.6%)	25 (34.2%)	1 (33.3%)	30 (33.3%)
	Control group	Colchicine	6 (48.9%)	37 (50.7%)	2 (67.7%)	45 (50.0%)
Total			14 (100.0%)	73 (100.0%)	3 (100.0%)	90 (100.0%)

According to Table 4, GH levels were within the normal range for patients using anakinra (*n* = 15) and canakinumab (*n* = 22) in the experimental group, as well as for those using colchicine (*n* = 33) in the control group. Similarly, IGF‐1 levels were observed to be in the normal range for patients using anakinra (*n* = 15) and canakinumab (*n* = 29) in the experimental group, as well as for those using colchicine (*n* = 42) in the control group.

**Table 4:** Comparison of growth hormone and IGF‐1 levels according to the reference interval of measurements.

Growth hormone and IGF‐1						
Value range			Low	Medium	High	Total
GH	Experimental group	Anakinra	0 (0.0%)	15 (21.4%)	0 (0.0%)	15 (16.7%)
		Canakinumab	8 (47.1%)	22 (31.4%)	0 (0.0%)	30 (33.3%)
	Control group	Colchicine	9 (52.9%)	33 (47.1%)	3 (100.0%)	45 (50.0%)
Total			17 (100.0%)	70 (100.0%)	3 (100.0%)	90 (100.0%)
IGF‐1	Experimental group	Anakinra	0 (0.0%)	15 (17.4%)	0 (0.0%)	15 (16.7%)
		Canakinumab	1 (33.3%)	29 (33.7%)	0 (0.0%)	30 (33.3%)
	Control group	Colchicine	3 (66.7%)	42 (48.8%)	1 (100.0%)	45 (50.0%)
Total			4 (100.0%)	86 (100.0%)	1 (100.0%)	90 (100.0%)

According to the experimental group in Table 5, PRL levels were in the normal range in patients who used anakinra (*n* = 11) and canakinumab (*n* = 24) and PRL levels were in the normal range in patients who used colchicine (*n* = 39) in the control group.

**Table 5:** Comparison of prolactin levels according to the reference interval of measurements.

Prolactin					
Value range			Medium	High	Total
PRL	Experimental group	Anakinra	11 (14.9%)	4 (25.0%)	15 (16.7%)
		Canakinumab	24 (32.4%)	6 (37.5%)	30 (33.3%)
	Control group	Colchicine	39 (52.7%)	6 (37.5%)	45 (50.0%)
Total			74 (100.0%)	16 (100.0%)	90 (100.0%)

When Table 6 is analyzed, significant differences were found in some hormone levels between anakinra–canakinumab and colchicine groups. In this context, statistically significant differences were observed between the groups in FSH, testosterone, and GH hormones. FSH levels were significantly lower in the experimental group compared to the control group (*p* = 0.032), and this difference showed a moderate effect size with Cohen’s d = −0.46. Similarly, GH levels were significantly lower in the anakinra–canakinumab group than in the colchicine group (*p* = 0.028), and the effect size of this difference was again moderate with Cohen’s d = −0.47. Testosterone hormone level was significantly higher in the experimental group (*p* = 0.035), and a high effect size was reached with Cohen’s d = 0.76.

**Table 6:** Comparison of pituitary and target organ hormone measurement averages for experimental (anakinra–canakinumab) and control groups.

Target organ hormone	Groups	*n*	Mean	s.d.	*t*	*p*	Cohen’s d
TSH	Experiment (anakinra–canakinumab)	45	2.48	1.55	1.531	0.129	—
	Control (colchicine)	45	2.03	1.22			
FT4	Experiment (anakinra–canakinumab)	45	1.21	0.18	1.566	0.121	—
	Control (colchicine)	45	1.14	0.23			
FT3	Experiment (anakinra–canakinumab)	45	3.11	0.50	−0.038	0.970	—
	Control (colchicine)	45	3.11	0.43			
FSH	Experiment (anakinra–canakinumab)	**45**	**6.40**	**7.75**	**−2.203**	**0.032** ^**∗**^	**−0.46**
	Control (colchicine)	**45**	**15.38**	**26.23**			
LH	Experiment (anakinra–canakinumab)	45	9.27	6.13	−1.271	0.209	—
	Control (colchicine)	45	12.45	15.57			
T TEST	Experiment (anakinra–canakinumab)	**21**	**4.99**	**1.49**	**2.195**	**0.035** ^**∗**^	**0.76**
	Control (colchicine)	**14**	**3.86**	**1.48**			
Estradiol	Experiment (anakinra–canakinumab)	24	164.31	191.82	1.695	0.101	—
	Control (colchicine)	32	94.07	76.82			
ACTH	Experiment (anakinra–canakinumab)	45	23.78	12.18	1.741	0.085	—
	Control (colchicine)	45	18.84	14.64			
Cortisol	Experiment (anakinra–canakinumab)	45	11.29	4.01	0.510	0.611	—
	Control (colchicine)	45	10.80	4.97			
GH	Experiment (anakinra–canakinumab)	**45**	**0.59**	**1.37**	**−2.239**	**0.028** ^**∗**^	**−0.47**
	Control (colchicine)	**45**	**1.84**	**3.48**			
IGF‐1	Experiment (anakinra–canakinumab)	45	144.87	47.79	1.322	0.190	—
	Control (colchicine)	45	130.85	52.66			
PRL	Experiment (anakinra–canakinumab)	45	15.51	10.41	0.476	0.635	—
	Control (colchicine)	45	14.50	9.59			

*Note:* Bold values indicate statistically significant differences between groups (*p* < 0.05).

^∗^ = *p* < 0.050; *p* > 0.050.

However, there was no statistically significant difference between the two groups in TSH, FT3, FT4, LH, estradiol, ACTH, cortisol, IGF‐1, and PRL hormones (*p* > 0.05). Although results close to significance were obtained especially in ACTH (*p* = 0.085) and estradiol (*p* = 0.101) hormones, these differences remained below the significance level. In conclusion, the findings show that anakinra–canakinumab treatment may be effective on some target organ hormones, especially on FSH, T TEST, and GH hormones, and it seems to have a significant and clinically moderate to high effect.

When Table 7 is analyzed, significant differences were observed in FSH and GH hormone levels between anakinra and colchicine groups. FSH level was significantly lower in the anakinra group compared to the colchicine group (*p* = 0.008), indicating a moderate effect size with Cohen’s d = −0.48. Similarly, GH level was significantly lower in the anakinra group (*p* = 0.005), indicating a moderate effect with Cohen’s d = −0.52. Regarding other hormones, TSH, FT3, FT4, LH, testosterone, estradiol, ACTH, cortisol, IGF‐1, and PRL levels were not significantly different between the groups (*p* > 0.05). Although results close to significance were obtained especially in estradiol (*p* = 0.061) and ACTH (*p* = 0.079) levels, these differences remained below the statistical limit. In conclusion, the study showed that anakinra treatment had different effects on some target organ hormones, especially on FSH and GH hormones with significant and moderate effects.

**Table 7:** Comparison of pituitary and target organ hormone measurement averages for colchicine and anakinra.

Target organ hormone	Groups	*n*	Mean	s.d.	*t*	*p*	Cohen’s d
TSH	Colchicine	45	2.03	1.22	−1.112	0.271	—
	Anakinra	15	2.49	1.82			
FT4	Colchicine	45	1.14	0.23	−0.291	0.772	—
	Anakinra	15	1.16	0.19			
FT3	Colchicine	45	3.11	0.43	0.173	0.863	
	Anakinra	15	3.09	0.49			
FSH	Colchicine	**45**	**15.38**	**26.23**	**2.791**	**0.008** ^**∗**^	**−0.48**
	Anakinra	**15**	**4.40**	**1.61**			
LH	Colchicine	45	12.45	15.57	0.751	0.456	—
	Anakinra	15	9.28	8.22			
T TEST	Colchicine	14	3.86	1.48	−1.511	0.147	—
	Anakinra	7	4.87	1.32			
Estradiol	Colchicine	32	94.07	76.82	−2.212	0.061	—
	Anakinra	8	298.50	258.51			
ACTH	Colchicine	45	18.84	14.64	−1.787	0.079	—
	Anakinra	15	26.56	14.03			
Cortisol	Colchicine	45	10.80	4.97	−0.546	0.587	—
	Anakinra	15	11.60	4.59			
GH	Colchicine	**45**	**1.84**	**3.48**	**2.982**	**0.005** ^**∗**^	**−0.52**
	Anakinra	**15**	**0.27**	**0.31**			
IGF‐1	Colchicine	45	130.85	52.66	0.576	0.568	—
	Anakinra	15	124.86	26.46			
PRL	Colchicine	45	14.50	9.59	0.033	0.974	—
	Anakinra	15	14.42	6.98			

*Note:* Bold values indicate statistically significant differences between groups (*p* < 0.05).

^∗^ = *p* < 0.50; *p* > 0.050.

When Table 8 was analyzed, no significant differences were found between colchicine and canakinumab in terms of thyroid functions (TSH, FT4, and FT3; *p* > 0.050), as well as in FSH, LH, T TEST, and estradiol measurements (FSH, LH, T TEST, and estradiol; *p* > 0.050). Similarly, no significant differences were observed in ACTH and cortisol measurements (ACTH and cortisol; *p* > 0.050), as well as in GH and IGF‐1 levels (GH and IGF‐1; *p* > 0.050). Lastly, PRL levels did not show any significant differences between the two groups (PRL; *p* > 0.050).

**Table 8:** Comparison of pituitary and target organ hormone measurement averages for colchicine and canakinumab.

	Groups	*n*	Mean	s.d.	*t*	*p*
TSH	Colchicine	45	2.03	1.22	−1.444	0.153
	Canakinumab	30	2.48	1.43		
FT4	Colchicine	45	1.14	0.23	−1.877	0.064
	Canakinumab	30	1.23	0.17		
FT3	Colchicine	45	3.11	0.43	−0.053	0.985
	Canakinumab	30	3.12	0.52		
FSH	Colchicine	45	15.38	26.23	1.872	0.066
	Canakinumab	30	7.40	9.31		
LH	Colchicine	45	12.45	15.57	1.274	0.208
	Canakinumab	30	9.27	4.94		
T TEST	Colchicine	14	3.86	1.48	−2.031	0.053
	Canakinumab	14	5.05	1.61		
Estradiol	Colchicine	32	94.07	76.82	−0.119	0.906
	Canakinumab	16	97.22	104.06		
ACTH	Colchicine	45	18.84	14.64	−1.129	0.263
	Canakinumab	30	22.39	11.13		
Cortisol	Colchicine	45	10.80	4.97	−0.310	0.758
	Canakinumab	30	11.14	3.76		
GH	Colchicine	45	1.84	3.48	1.816	0.074
	Canakinumab	30	0.75	1.65		
IGF‐1	Colchicine	45	130.85	52.66	1.929	0.058
	Canakinumab	30	154.88	53.07		
PRL	Colchicine	45	14.50	9.59	−0.624	0.535
	Canakinumab	30	16.05	11.83		

*Note: p* > 0.050.

## 4. Discussion

In this paper, hormone levels such as ACTH, cortisol, FSH, LH, PRL, GH, and IGF‐1 were compared between FMF patients receiving IL‐1 antagonist therapy and those not receiving it. The findings are significant as they represent one of the first studies to evaluate the effects of IL‐1 antagonist therapies (anakinra and canakinumab) on hormone levels.

In a systematic review by Moutschen et al. assessing the efficacy of IL‐1 antagonist therapy in FMF patients, it was reported that anakinra treatment was evaluated in 22 publications including 64 patients, while canakinumab was assessed in 8 studies involving 40 patients. Serious adverse effects such as injection site reactions and interstitial pneumonia were observed in patients using anakinra, whereas no serious side effects were reported for canakinumab [20]. Ali Şahin et al. also reported no significant differences in adverse effects between patients receiving anakinra and canakinumab [21].

The effects of IL‐1 on pituitary hormones have been extensively studied in the literature. The effects of endotoxins on pituitary hormones have long been recognized. Bernton et al. demonstrated that IL‐1 stimulates the release of ACTH, LH, and TSH from pituitary cells [22]. Rivier et al. found that IL‐1, IL‐6, and TNF‐α activate the HPA axis by increasing CRF secretion, playing a crucial role in the release of pituitary hormones [23]. Jeffrey Flier et al. showed that these cytokines synergistically stimulate the HPA axis through a potent mechanism [24]. Cytokines such as IL‐1 and TNF‐α have been shown to activate the NF‐κB pathway, increasing inflammatory responses and exerting inhibitory effects on pituitary hormones [25]. Parnet et al. demonstrated that IL‐1 plays a role in regulating pituitary hormones by activating the NF‐κB pathway [26].

Rasmussen et al. demonstrated that IL‐1 suppresses thyroid cell function by reducing TSH secretion [27]. Dubuis et al. reported that IL‐1 decreases plasma thyroid hormone levels, and its effects on the thyroid occur through inhibition of TSH secretion at the pituitary level [28]. Kang et al. showed that IL‐1β suppresses GnRH expression, leading to reduced LH levels [29]. Herman et al. found that IL‐1 decreases the biosynthesis of LH and FSH and reduces the expression of GnRH receptors [30]. Tremellen et al. demonstrated that exposure to endotoxins suppresses testosterone production by Leydig cells, leading to testosterone deficiency [31]. Studies on IGF‐1 and GH levels have shown that inflammatory cytokines exert inhibitory effects on these hormones. Jason Connor et al. demonstrated that proinflammatory cytokines disrupt IGF‐1 signaling, thereby inhibiting cellular proliferation [32]. IL‐1 has been specifically noted to suppress the biosynthesis of GH and IGF‐1, affecting growth functions [25].

In this paper, no significant differences were found in ACTH, cortisol levels, and thyroid function tests between FMF patients who had been using IL‐1 antagonists for at least 6 months and those who had not (*p* > 0.050). Similarly, no differences in ACTH, cortisol levels, and thyroid function tests were observed between patients treated with anakinra and canakinumab. However, some patients taking IL‐1 antagonists and colchicine have been noted to have low cortisol levels. A Synacthen test was performed in these patients, and adrenal insufficiency was not detected. This finding is consistent with the existing literature [33].

## 5. Conclusion

In this paper, a significant difference was found in FSH, T TEST, and GH levels between the control and experimental groups that received and did not receive IL‐1 antagonist (*p* < 0.050). On the other hand, significant differences were found between colchicine and anakinra in terms of FSH and GH (*p* < 0.050). Between the control and experimental groups, FSH was found to be higher in the control group receiving colchicine. This finding may also be due to the high number of postmenopausal patients in this group. Nevertheless, we can say that IL‐1 antagonists do not lower FSH.

Between the control group and the experimental group, which received or did not receive IL‐1 antagonist, the T TEST value was found to be higher in the group receiving IL‐1 antagonist. There is no significant difference in LH and FSH of these patients. This result suggests that the effects of the inflammatory response on testosterone production may differ depending on the treatment.

In this paper, GH levels were found to be higher in the control group receiving colchicine. Without dynamic testing (glucagon stimulation test or insulin tolerance test), it is not possible to comment on GH levels. An insulin tolerance test could have been performed in these patients. However, as they may have serious comorbidities, it was not performed in our study and patients were assessed clinically.

In conclusion, the effects of IL‐1 antagonist therapy on hormone levels in FMF patients were found to be limited. Given the known effects of IL‐1 on pituitary hormones, further research is required to understand the hormonal side effects of this therapy. Studies evaluating the effects of IL‐1 antagonist therapy on pituitary function are limited in the literature. Therefore, more studies are needed to evaluate the long‐term hormonal effects of IL‐1 antagonist therapy.

**Nomenclature**
FMF: Familial Mediterranean fever
IL‐1: Interleukin‐1
TSH: Thyroid‐stimulating hormone
FT3: Free triiodothyronine
FT4: Free thyroxine
ACTH: Adrenocorticotropic hormone
LH: Luteinizing hormone
FSH: Follicle‐stimulating hormone
GH: Growth hormone
IGF‐1: Insulin‐like growth factor‐1
PRL: Prolactin
MSH: Melanocyte‐stimulating hormone
ADH: Antidiuretic hormone
T TEST: Total testosterone
HPA: Hypothalamic–pituitary–adrenal
CUBAP: Scientific Research Projects Coordination Unit of Sivas Cumhuriyet University

## Ethics Statement

This study was approved by the Ethics Committee of Sivas Cumhuriyet University Faculty of Medicine on 16.02.2021 (Decision No. 2021‐02/05).

## Disclosure

This paper is derived from the medical residency thesis of the first author under the supervision of the second and third authors. All scientific responsibility rests with the authors, and the final version of the manuscript was thoroughly reviewed and approved by the authors.

## Conflicts of Interest

The authors declare no conflicts of interest.

## Author Contributions

Conceptualization: F.A.K., B.S., and A.S.; data curation: F.A.K.; formal analysis: F.A.K. and B.S.; funding acquisition: F.A.K.; investigation: F.A.K., B.S., and A.S.; methodology: F.A.K., B.S., and A.S.; project administration: B.S. and A.S.; resources: F.A.K. and B.S.; software: F.A.K. and B.S.; supervision: B.S. and A.S.; validation: B.S. and A.S.; visualization: F.A.K., B.S., and A.S.; writing–original draft: F.A.K., B.S., and A.S.; and writing–review and editing: F.A.K., B.S., and A.S..

## Funding

This study was funded by Sivas Cumhuriyet Üniversitesi, T‐2021‐930.

## Data Availability

The data that support the findings of this study are available from the corresponding author upon reasonable request.
